# New insights into population structure, demographic history, and effective population size of the critically endangered blue shark *Prionace glauca* in the Mediterranean Sea

**DOI:** 10.1371/journal.pone.0305608

**Published:** 2024-06-17

**Authors:** Violaine Dolfo, Emilie Boissin, Matthieu Lapinski, Serge Planes

**Affiliations:** 1 CRIOBE UAR3278, PSL Research University: EPHE–UPVD–CNRS, Perpignan, France; 2 Laboratoire d’Excellence « CORAIL », PSL Research University: EPHE–UPVD–CNRS, Perpignan, France; 3 Association AILERONS, Université de Montpellier, Montpellier, France; Shark Research Institute, UNITED STATES

## Abstract

The blue shark, *Prionace glauca*, is the most abundant pelagic shark in the open ocean but its vulnerability remains poorly understood while being one of the most fecund sharks. In the Mediterranean Sea, the blue shark is listed as Critically Endangered (CR) by the International Union for Conservation of Nature. The species is facing a strong decline due to fishing, and scientific data regarding its genetic structure and vulnerability are still lacking. Here, we investigated the genetic diversity, demographic history, and population structure of the blue shark within the Mediterranean Sea, from samples of the Gulf of Lion and Malta, using sequences of the mtDNA control region and 22 microsatellite markers. We also compared our mitochondrial data to previous studies to examine the Atlantic-Mediterranean population structure. We assessed the blue shark’s genetic vulnerability in the Mediterranean basin by modelling its effective population size. Our results showed a genetic differentiation between the Atlantic and the Mediterranean basins, with limited gene flow between the two areas, and distinct demographic histories making the Mediterranean population an independent management unit. Within the Mediterranean Sea, no sign of population structure was detected, suggesting a single population across the Western and Central parts of the sea. The estimated effective population size was low and highlighted the high vulnerability of the Mediterranean blue shark population, as the estimated size we calculated might not be sufficient to ensure the long-term persistence of the population. Our data also provide additional evidence that the Gulf of Lion area acts as a nursery for *P*. *glauca*, where protection is essential for the conservation strategy of the species in the Mediterranean.

## Introduction

A good understanding of population ecology and genetics is essential for species conservation. This requires information about population delimitation and structuration, dynamics and size, as well as reproductive strategy. This information is challenging to obtain for highly mobile marine species due to the difficulty of observation and sampling [1]. However, genetic data have contributed to detecting genetically distinct populations and connectivity and understanding population dynamics and genetic vulnerability [2]. One parameter of particular importance is the effective population size (*Ne*), defined as the size of an idealized population, giving the same rate of genetic drift as observed in the population of interest [3]. The effective population size provides information regarding how quickly genetic diversity may be lost [4,5], which in turn may lead to a reduction of the population’s adaptation capabilities and threaten its survival in a rapidly changing environment [6].

The Mediterranean Sea covers only 1% of the oceans’ surface, while it hosts about 7% of the total marine biodiversity, with numerous endemic species [7]. It is also one of the most populated basins with about half a billion inhabitants driving coastal habitat loss, overexploitation, and pollution, all affecting biodiversity [8], and making the Mediterranean Sea a conservation priority for national and international agencies [9]. Chondrichthyans (cartilaginous fishes) are one particularly vulnerable group, partly because their life history traits (i.e. slow growth, late maturity, and low fecundity) do not favour fast adaptation to the environmental pressures and induce slow recovery of depleted populations [10]. According to the International Union for Conservation of Nature (IUCN), the Mediterranean region has the highest percentage of threatened chondrichthyans in the world: 53% of the species are threatened with extinction [11]. The main cause of the decline is overfishing, including by-catch. Among these species, the blue shark, *Prionace glauca*, is no exception.

The blue shark has a circumglobal distribution in temperate and tropical waters and is the most abundant pelagic shark in the open ocean [12]. It is the only shark of the *Prionace* genus, and has one of the highest fecundity (30 pups on average) and earliest maturity (4 to 6 years) of the Carcharhinidae family, leading to a generation time of approximately eight years [13]. This large species (Total Length > 300 cm) is highly migratory and can cover up to 10’000 km including transoceanic movements [13]. Across the oceans, blue sharks are segregated by sex and reproductive stages, and exhibit migrations reflecting both prey availability and reproductive cycle [13]. Philopatry to foraging sites and nursery grounds has been observed in the Atlantic Ocean [14,15]. Nurseries occur in both coastal open areas [16] and in pelagic habitats [17]. Regarding conservation status, the Mediterranean blue shark is listed as “Critically Endangered” on the Red List of the IUCN, while it is listed as “Near Threatened” throughout the rest of its range [18]. Although it is one of the most abundant sharks in the region, this status is based on an estimated population decline of 90% over three generations [18].

As an important resource for the fisheries, blue shark stock structure assessments based on genetic approaches have been carried out both at regional and global scales across its range. However, they provide conflicting views of the genetic connectivity of global populations. At a global scale, no genetic structure has been detected, suggesting a global panmixia [19]. More regionally, genetic homogeneity is observed across the North Pacific [20], while the population structure of the Atlantic blue shark remains unclear. Significant structuring between nurseries from North-East and South-East Atlantic was detected from mitochondrial and microsatellite markers [21] but a panmixia across the whole Atlantic is suggested from the same dataset enriched with samples from Brazilian nurseries [1]. For management purposes, the Mediterranean blue shark is considered a distinct stock [22]. However, this delineation was challenged by Leone *et al*. [23,24] who revealed some degrees of genetic connectivity between Western Mediterranean and adjacent Atlantic populations based on Single Nucleotide Polymorphism (SNP) markers. They suggest that the Mediterranean serves as a nursery for the Atlantic blue shark population, but also reveal weak but significant genetic variation between Eastern and Western Mediterranean blue shark populations. More recently, using genome-wide SNPs, Nikolic *et al*. [25] revealed a clear split between samples from the Indo-Pacific and samples from the Atlantic and also found a subtle but significant structure between Atlantic and Mediterranean Sea populations. The blue shark population structure in the Mediterranean Sea seems complex and its long-term genetic vulnerability remains unknown, although this basin appears to be both an important ecological area and a zone of threat for this species. Blue sharks are indeed frequently caught by various fishing gears in the Mediterranean [26], and catch data are still under-reported to date [22].

In this study we aim to characterize the blue shark population in the Mediterranean Sea and more specifically in its North Western part, the Gulf of Lion, by i) exploring its genetic and demographic structure and its evolutionary history within the Mediterranean, ii) exploring the genetic differentiation between blue shark from the Mediterranean Sea and the Atlantic, and iii) assessing its genetic vulnerability in the Mediterranean Sea through estimations of its genetic diversity and effective population size.

## Material and methods

### Sample collection

**Fig 1:**
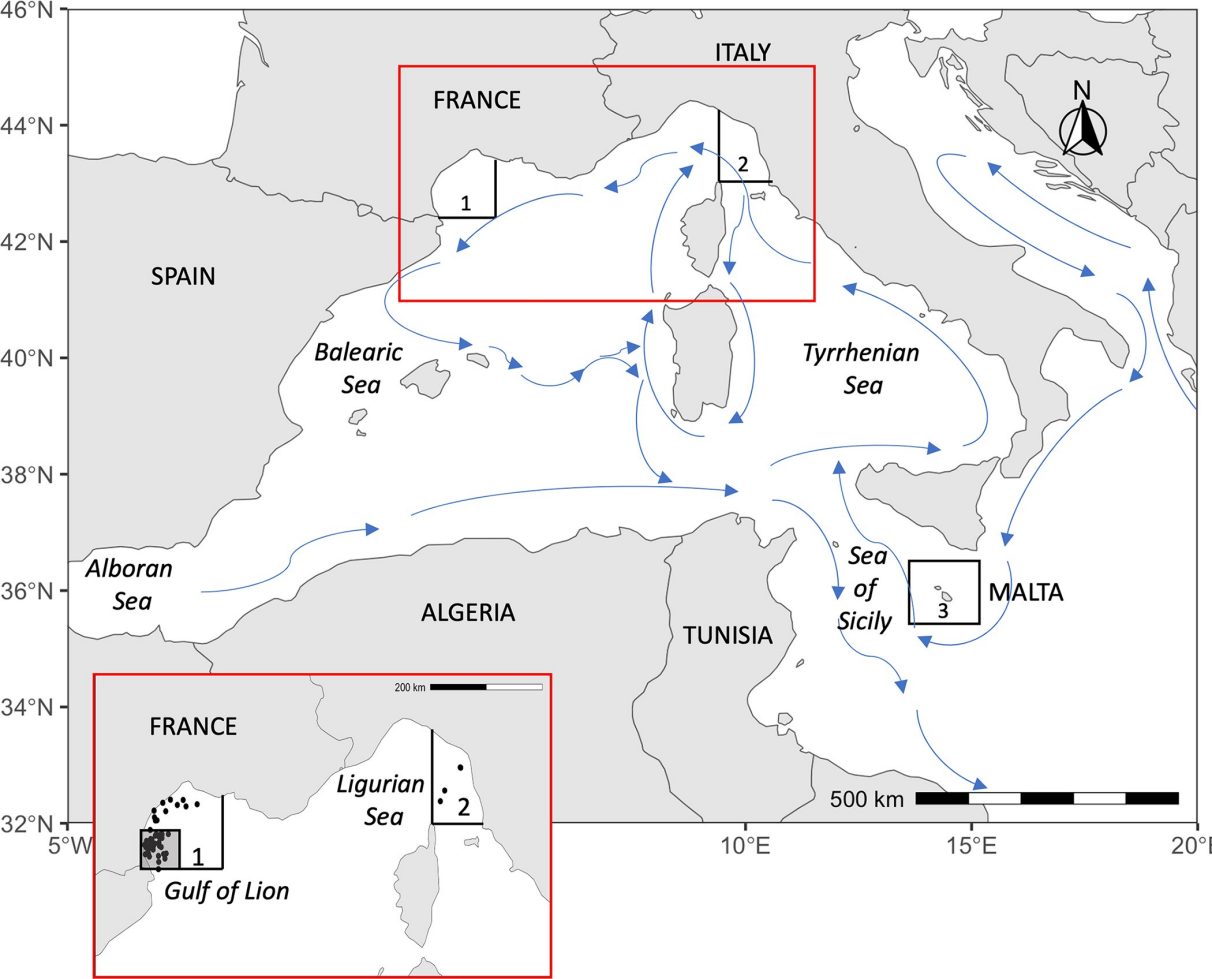
Sampling locations. Sampling locations of blue sharks in the Gulf of Lion (1), the Ligurian Sea (2), and Malta (3) and main currents in the Western Mediterranean basin. The zoomed map represents the GPS position of individuals sampled in Zone 1 and Zone 2 and the Marine Park of the Gulf of Lion (shaded area). GPS position for sharks in zone 3 was not available. The map was created using the R software and the publicly available map dataset *Natural Earth*.

Blue shark muscle tissues were collected at two locations in the Mediterranean Sea (Fig 1). Blue shark fishing is unregulated in the Mediterranean Sea and requires no specific permit. In the Gulf of Lion and the Ligurian Sea (GUL), recreational fishermen and the Association Ailerons (France) collected blue shark samples opportunistically using no-kill line fishing between June and September, from 2012 to 2018, as part of a citizen science program (Fig 1). Biopsies of approximately 1 cm were taken on the dorsal fin (free rear tip) before releasing the animal. Non-lethal fishing techniques were used and the best handling practices of the Food and Agriculture Organization of the United Nations (FAO) were followed to reduce post-sampling stress and maximize survival. Total length (TL), sex, and GPS coordinates were recorded when possible. Samples from the Ligurian Sea were pooled with those from the Gulf of Lion due to the low sampling size in the former location (n = 3). In Malta (MAL), blue shark samples were collected by the Association Sharklab on dead sharks from industrial fishing vessels after landing. TL and sex were recorded when possible. Tissue samples were stored at room temperature in 90% ethanol until processing.

A total of 192 individuals were sampled (GUL: N = 112, MAL: N = 80), 167 were measured (GUL: N = 87, MAL: N = 80) and 123 were sexed *in situ* by fishermen and NGO volunteers (GUL: N = 44, MAL: N = 79) (S1 Table). The age of each individual was estimated from TL based on the von Bertalanffy growth model: $L_{t}=L_{\infty }(1-e^{-k({t-t}_{0})})$, where *L*_*t*_ is the length at age *t*, *L*_*∞*_ the asymptotic length, *k* is the growth parameter, and *t*_*0*_ is the theoretical age at which the length is equal to zero. The values of *L*_*∞*_ = 401.55 cm, k = 0.13, *t*_*0*_ = -0.62 years used in this study were calculated by Megalofonou et al. (16] for Mediterranean blue sharks. The difference in length distribution between GUL and MAL was tested with a Student’s test on the mean length from both locations.

### DNA extraction and molecular analyses

DNA was extracted using the QIAamp 96 DNA QIAcube HT Kit and the QIAcube extraction robot (QIAGEN GmbH, Hilden, Germany) following the manufacturer’s protocol. The first step was modified as follows: 5mm^2^ of tissue samples were placed in 200μL of Proteinase K solution (1 volume for 3 volumes of buffer VXL, QIAGEN GmbH) and incubated at 55°C for 1h40.

A 900 base pairs (bp) fragment of the mitochondrial control region CR was amplified with the blue shark specific primers RT-ProL (5’-AAGGAGGATCAAACTCCTGCC-3’) and RT-12SH (5’-ACTAAGGCTAGGACCAAACC-3’) designed by Taguchi et al. [27]. The PCR reaction was performed in a final volume of 26μL containing 2.5μL of Buffer 10X, 2μL of MgCl_2_ (25mM), and 0.125μL of Taq polymerase (5U/μL) from the Taq PCR Core Kit (QIAGEN GmbH), 2.5μL of dNTP mix (100mM), 0.6μL of each primer (10μM, Eurofin Genomics, Paris, France), 14.175μL of SIGMA water, and 3.5μL of template DNA (10–30μM). The temperature profile followed the protocol described in Taguchi et al. [27] modified with 40 cycles of amplification instead of 27 cycles. PCR amplicons were sequenced using the external service provider GenoScreen (Lille, France).

Thirty microsatellite loci developed specifically for *P*. *glauca* were obtained from Fitzpatrick et al. [28], Mendonça et al. [29], and Taguchi et al. [30], but only 25 loci were consistently amplified in our samples. Five multiplex reactions were carried out at different annealing temperatures in a final volume of 10μL with the Type-it Microsatellite PCR Kit (QIAGEN GmbH) (S2 Table). The final reaction volume contained 4μL of Multiplex PCR Master Mix 2X (QIAGEN GmbH), 1μL of primer mix, 4μL of RNAse-free water, and 1μL of DNA. Polymerase chain reaction amplifications used the fluorescently labelled forward primer of each locus (e.g. TET, FAM, TAMRA, CY5; Macrogen Europe, Amsterdam, Holland). The thermal cycling profile involved one cycle of Taq activation for 5 min at 95°C followed by 40 cycles of denaturation for 30 s at 95°C, annealing for 90 s at optimal temperature (S2 Table), and extension for 30 s at 72°C; and a final extension step for 30 min at 60°C. PCR products were analysed using the external service provider GenoScreen (Lille, France). GeneMapper software v3.7 (Applied Biosystems) was used to score individual genotypes manually.

### Genetic diversity, population structure, and demographic history analyses

#### Mitochondrial control region marker

Obtained sequences of the mitochondrial control region (CR) were aligned with homologous CR sequences of *P*. *glauca* available in GenBank using the ClusterW algorithm implemented in MEGA v7.0 [31] (S3 Table). For comparison with other studies, the fragments were truncated to 720 bp. All the mitochondrial sequences produced in this study were deposited in GenBank, under the accession numbers PP797150—PP797299. Mitochondrial DNA diversity indices were calculated with the DnaSP software v5.10.01 [32] including the total number of haplotypes (H), polymorphic sites (S), haplotype diversity (h), and nucleotide diversity (π). The diversity indices were calculated for the following datasets: Gulf of Lion (GUL) and Malta (MAL) (data from this study only, n = 150), Mediterranean Sea (MED, data from this study combined with those from Leone et al. [24] (n = 131)), and Atlantic (ATL, data from Leone et al. [24] (n = 39), Veríssimo et al. [1] (n = 273), Ferrette et al. unpublished (KY994016-KY994042, MH085076-MH085080, MH806840-MH806841 (n = 108)) (S1 Fig and S3 Table). The spatial distribution of haplotypes was explored with a Median Joining Haplotype Network [33] as implemented in the PopART software [34]. The network was built with all the sequences from the Atlantic Ocean (n = 420) and the Mediterranean Sea (n = 281) (combined dataset). To further investigate the population structure, the fixation index F_st_ and pairwise φ_st_ distances were calculated between the Atlantic and the Mediterranean. F_st_ was calculated using the package *hierfstat* v0.5–11 [35] in R [36], and the 95% confidence interval was computed with 100 bootstrap permutations. Pairwise φ_st_ was calculated with the R package *haplotypes* v1.1.3.1 [37] and 100 permutations.

To investigate demographic history at different scales within the Mediterranean Sea and the Atlantic Ocean, CR sequences from GUL and MAL were first pooled together (GUL/MAL). Then CR sequences from other Mediterranean regions were added to the dataset to determine demographic history at the basin’s scale (MED). Neutrality tests Fu and Li’s D and F [38] implemented in DnaSP were carried out on both datasets (GUL/MAL and MED). Significantly negative values indicate past population expansion, while positive values represent a genetic bottleneck. Additionally, the historical demographic trend of the two datasets was investigated using coalescent analysis with the Bayesian Skyline Plot (BSP) framework implemented in BEAST v1.8.4 [39,40] and summarized with Tracer v1.7.1 [41]. The best nucleotide substitution model was determined using MEGA v7.0. The HKY model with 4 gamma categories was then used, with a normal molecular clock distribution of 0.62% (Confidence Interval: 0.20%) of mutations per site per million years as prior. The molecular clock was estimated as an average for sharks based on the Isthmus of Panama biogeographical calibration on four shark species [42]. Three MCMC chains of 10 million steps logged every 100 steps were run with BEAST v1.8.4 and combined with LogCombiner v1.10.4 [43] with 1 million burn-in steps, ensuring a sufficient effective sampling size (ESS>200) as advised by the authors. The same analyses were carried out with the sequences from ATL to compare the historical evolution between the two basins.

#### Microsatellite markers

Microsatellite genotypes were checked for scoring errors, large allele dropout, and the presence of null alleles using MicroChecker v2.2.3 [44]. The complete matrix of genotypes is shown in S4 Table. The diversity indices were calculated for the dataset GUL and MAL, to compare the genetic diversity between the two locations. These include the mean number of alleles (Na) and rare alleles (Nar) per locus, and the expected and observed heterozygosities (He and Ho, respectively) calculated with GenAlEx v6.5 [45]. Additionally, the allelic richness (AR) was calculated with FSTAT v2.9.4 [46].

Genetic differentiation among sample collections within the Mediterranean (GUL, MAL) was explored through different approaches. First, the pairwise differentiation index G_st_ was estimated between the two regions based on the sampling location with GenAlEx v6.5. An exploratory Principal Coordinate Analysis (PCoA) was also performed in GenAlEx v6.5. Population structure within the Mediterranean was further explored through a Bayesian approach implemented in STRUCTURE v2.3.3 [47]. Ten independent series were run under the admixture ancestry model with correlated allelic frequencies for each assumed number of populations (K = 1–4). The sampling location was used as a prior to help distinguish weakly differentiated subpopulations with the use of the LOCPRIOR algorithm [48]. Each run was performed with an initial burn-in of 50,000 steps, followed by 400,000 MCMC (Marko chain Monte Carlo) repetitions. STRUCTURE HARVESTER v0.6.94 online [49] was used to assess K, the number of genetic populations that best fit the data, based on Maximum Likelihood [50].

Additionally, Colony v2.0.6.5 [51] was used to determine the family lineages between individuals (full-sib and half-sib relationships, parent-offspring relationships) and describe any fine-scale family structures. Adult individuals (TL > 202cm for males, and TL > 214 cm for females) [16] were considered as potential parents, and juveniles as potential offspring. The software was run three times with different random starting seeds to ensure the robustness of the analysis. For each run, three series were performed using the full-likelihood method with a high likelihood precision and a long-length run, allowing polygamy and inbreeding for both parents.

The contemporary effective population size (CNe) was calculated with individuals from GUL and MAL pooled together in one dataset. The Linkage Disequilibrium method as implemented in NeEstimator v2.1 [52] was chosen to allow comparison with CNe of blue shark populations from other areas [1,20] and thus assess the relative vulnerability of the Mediterranean blue shark (for a review of methods see [53,54]). Siblings detected with Colony were excluded from the analysis to remove potential family biases. The random mating model with a parametric 95% confidence interval was used. The P_CRIT_ parameter can be set at different thresholds to screen out rare alleles, which influence the value of CNe. Stable CNe indicates an isolated population while variations in CNe depending on P_CRIT_ suggest gene flows in the population history and/or the presence of first-generation immigrants [55,56]. Variations of CNe were investigated with P_CRIT_ = 0.01–0.02–0.05 (i.e. when removing alleles found with a frequency of 1%, 2%, and 5%), and without frequency restriction (P_CRIT_ = 0).

## Results

### Population characterisation within the Mediterranean

#### Demographic structure in the Gulf of Lion and Malta

**Fig 2:**
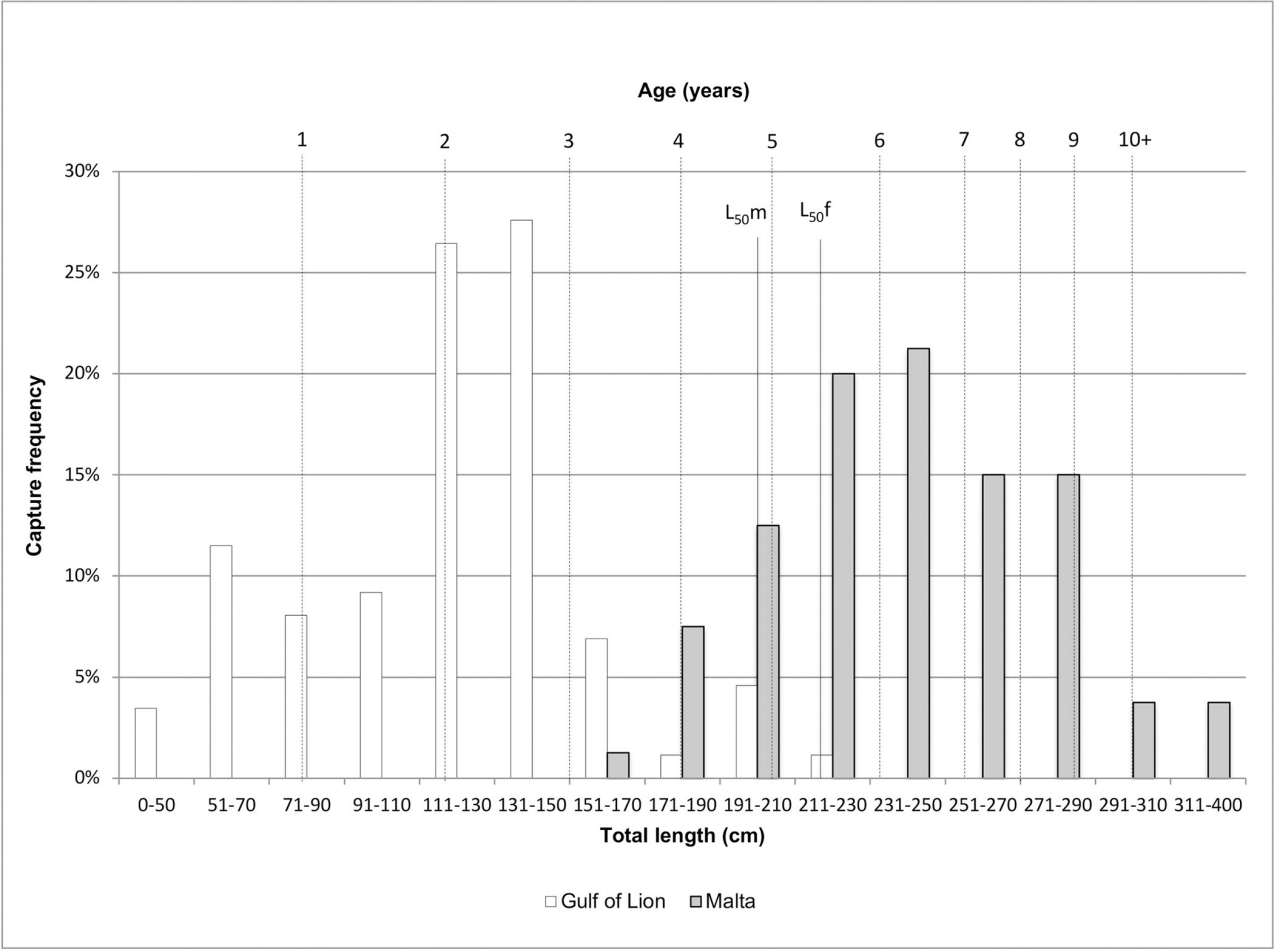
Length-frequency distribution and age estimation of 167 blue shark individuals sampled in the Gulf of Lion (white) and Malta (grey shade). Age was estimated according to Megalofonou and colleagues’ method and parameters [16]. L_50_m/f: length at 50% maturity for males and females, respectively.

The total length (TL) of 167 individuals from GUL and MAL varied from 35 cm (young of the year) to 355cm (more than 10 years old), with a clear difference in length distribution between the two locations (Fig 2, Student’s test, p-value < 0.001). In GUL, the median TL was 129.5cm (approx. 2 years) and sampled individuals reached a maximum of 212cm TL (approx. 5 years), which falls below the size at 50% of maturity (L_50_) for both males and females [16]. All individuals were therefore considered as juveniles. In MAL, the median TL was 233cm (approx. 6 years), with 76% of individuals considered as adults (i.e. TL>L_50_) and no individual shorter than 166 cm (aged less than 3 years). The sex ratio (male: female) was 1:1.82 in Malta (MAL) and 1:1 in the Gulf of Lion (GUL) but sex data was not available for 60.7% of the individuals in the Gulf of Lion.

#### Genetic diversity

The 150 mtDNA CR sequences obtained from GUL and MAL exhibited 12 polymorphic segregating sites, totalling 16 haplotypes for the truncated 720bp fragment later used for comparison with Atlantic and other Mediterranean haplotypes. The haplotype diversity was slightly lower in MAL (h = 0.759 ± 0.041) than in GUL (h = 0.805 ± 0.031) but the nucleotide diversity was similar between the two locations (MAL: π = 0.00314 ± 0.00022; GUL: π = 0.00330 ± 0.00020) (Table 1).

**Table 1:** Diversity indices from the mitochondrial and microsatellite datasets of blue sharks of the Gulf of Lion (GUL) and Malta (MAL).

	Mitochondrial diversity			Microsatellite diversity					
Location	N	h	π	N	Na	Nar	Ho	He	AR
GUL	75	0.805 ±0.031	0.0033 ±0.0002	109	10.0 ±1.7	5.3 ±1.2	0.661 ±0.038	0.687 ±0.037	9.45
MAL	75	0.759 ±0.041	0.0031 ±0.0002	80	10.2 ±1.4	5.6 ±1.1	0.667 ±0.034	0.687 ±0.036	10.04

N: number of individuals; h: haplotype diversity; π: nucleotide diversity; Na: mean number of alleles per locus; Nar: mean number of rare alleles per locus (allelic frequency <5%); Ho: observed heterozygosity; He: expected heterozygosity under Hardy-Weinberg expectations; AR: allelic richness.

At a microsatellite level, a total of 22 markers were successfully genotyped for 187 blue sharks. Null alleles were detected at loci A2ASY, Pgla05, CY92Z, DZONX, Pgla06, EHD08, and TB01, but none with a ratio higher than 0.1. They were therefore kept in further analyses. Stuttering errors were also detected at locus Pgla06, which was thus removed from the analysis. Loci TB15 and Pgla08 included more than 6% of missing data and were also removed, resulting in a final dataset cumulating 22 loci. Microsatellite genetic diversity was similar among samples from GUL and MAL. Total number of alleles per locus (Na) ranged from 2 to 36 (GUL: mean = 10.04 ± 1.65; MAL: mean = 10.18 ± 1.44), and number of rare alleles per locus (Nar) ranged from 0 to 30 (GUL: mean = 5.27 ± 1.21; MAL: mean = 5.59 ± 1.09). Levels of observed (Ho) and expected (He) heterozygosity and allelic richness (AR) were also similar in samples from both locations (Table 1).

#### Population structure within the Mediterranean basin

**Fig 3:**
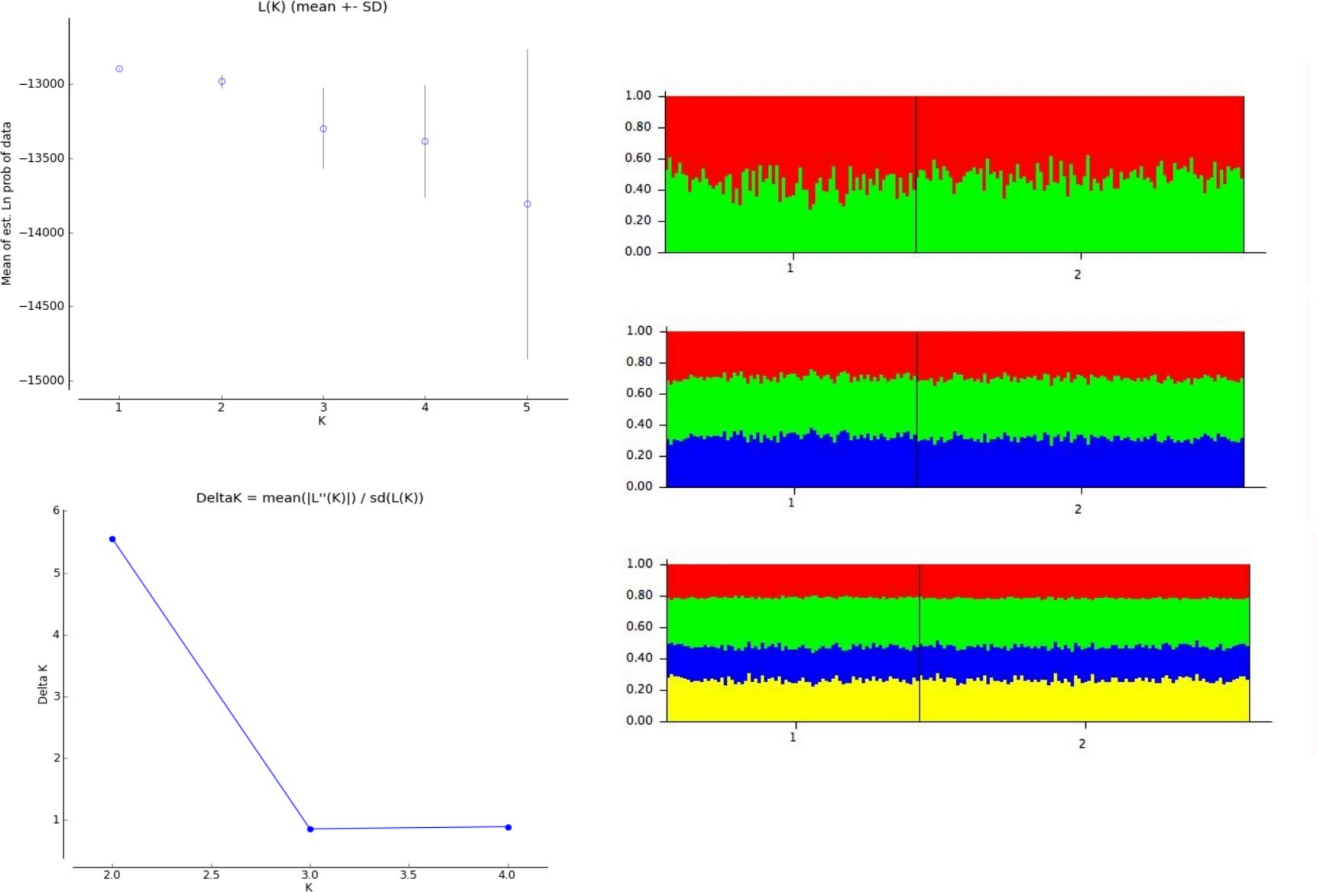
Bayesian clustering of blue shark individuals from STRUCTURE analysis after removing the full siblings from the analysis. A): Plot of the mean of estimated “log probability of data” for each value of K. B): DeltaK of Evanno’s method based on the rate of change in the log probability of data. C) Barplots for K from 2 to 4. Each individual is represented by a vertical bar partitioned into coloured sub-bars whose lengths are proportional to its estimated probability of membership for the K clusters. 1: individuals from Malta, 2: individuals from the Gulf of Lion.

The Bayesian clustering with STRUCTURE did not detect any genetic differentiation between samples from MAL and GUL. Noticeably, several small groups of 2 to 4 individuals showed a particular variance and appeared as outsiders on the bar plot; but these groups were not consistent among the different runs (S2 Fig). Interestingly, the family lineage analysis with Colony showed that these outsider groups were composed of full siblings (Table 2). They were more numerous in the Gulf of Lion (8 individuals forming 3 sibling groups) than in Malta (2 individuals forming 1 pair). When removing all the individuals but one in each group, blue shark genotypic data from Gul and MAL were best explained by one single genetic group (K = 1, which showed the highest likelihood and the lowest variance associated) and indicated no genetic structure between Malta and the Gulf of Lion (Fig 3). Additionally, the PCoA analysis did not show any significant differentiation between GUL and MAL (S3 Fig), and the pairwise differentiation index G_st_ was equal to zero (p_value = 0.19), indicating an absence of genetic structure between the two locations.

**Table 2:** Full-sib relationship and family cluster in the Mediterranean blue shark inferred with Colony v2.0.6.5.

Cluster #	Offspring ID1	Offspring ID2	Probability	Location
1	4APg	5APg	1	GUL
	14APg	9APg	0.998	
	14APg	4APg	0.997	
	14APg	5APg	0.993	
2	64Pg	65Pg	1	GUL
3	HPg	IPg	1	MAL
4	6APg	7APg	0.999	GUL

GUL: Gulf of Lion, MAL: Malta.

#### Demographic history and contemporary effective population size

**Fig 4:**
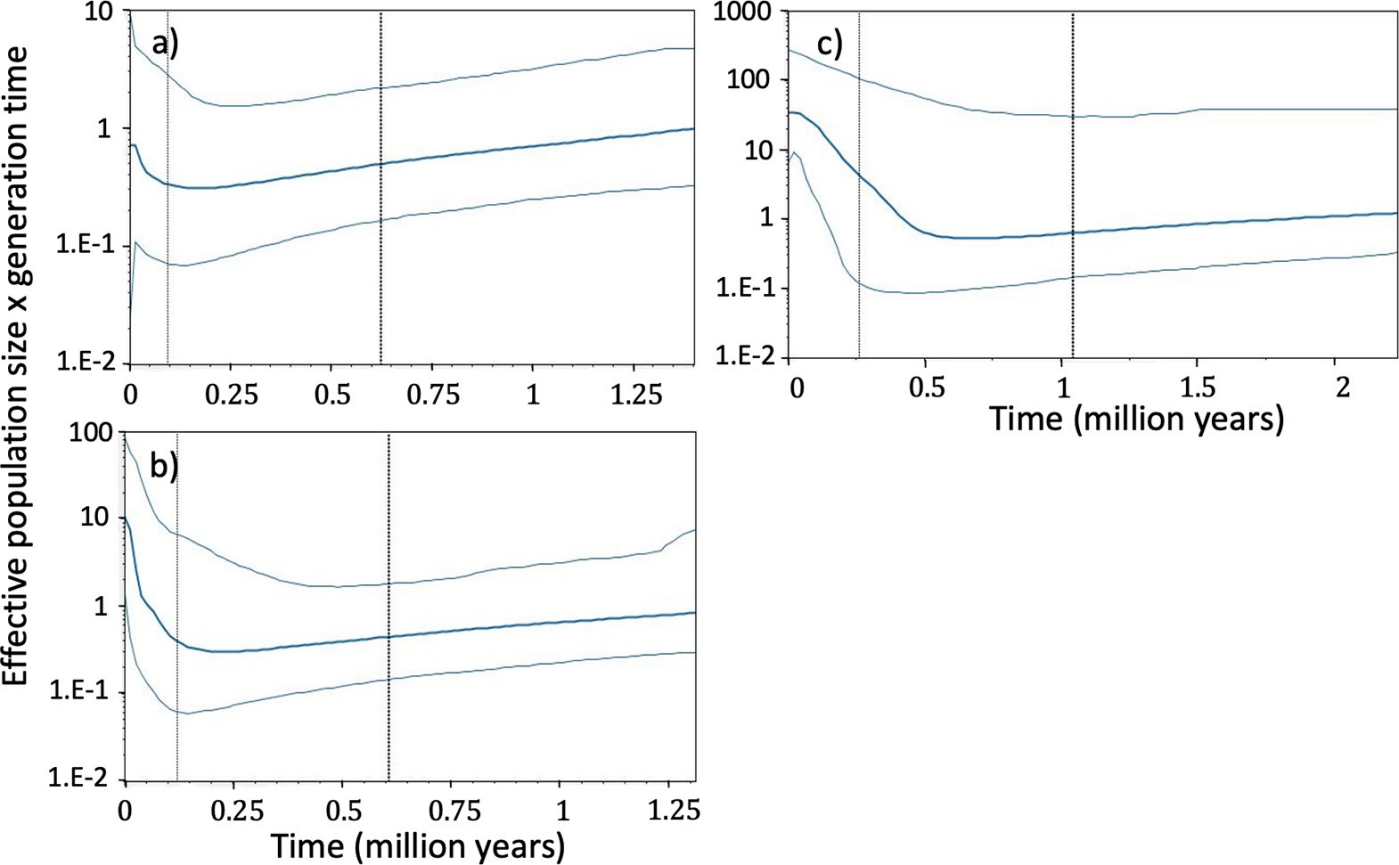
Bayesian Skyline Plot from a fragment of 720 bp of the mtDNA control region. a) Gulf of Lion and Malta, b) Mediterranean; c) Atlantic. The Y-axis indicates effective population size x generation time, while the X-axis indicates the mean time in millions of years before present. The thick line represents the median estimate and the thin lines represent the 95% confidence interval.

Regarding the historical population size modelled from mtDNA CR sequences, Fu and Li’s F and D tests did not detect any significant deviation from selective neutrality and population equilibrium either in the sampling areas (GUL/MAL) or in the Mediterranean (MED, p-value > 0.1). However, BSP analyses on mtDNA CR sequences suggested a population size increase in the Gulf of Lion and Malta; starting approx. 0.15 Mya (Fig 4A). When other sequences from the Mediterranean basin were included, a similar expansion was observed, but with greater intensity, suggesting no difference in the historical demographic trend at the local and global scale within the Mediterranean basin (Fig 4B).

**Fig 5:**
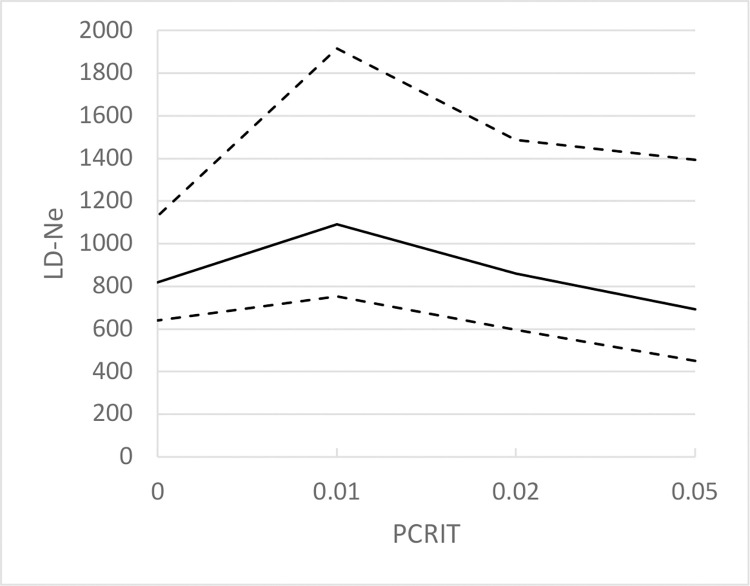
Variation of the value of the effective population size estimated with the linkage disequilibrium method in NeEstimator v2.1 depending on the number of rare alleles screened out (P_crit_ parameter).

The contemporary effective population size (CNe) based on microsatellite genotypes of individuals from GUL and MAL pooled together appeared stable depending on the P_CRIT_ value, suggesting population isolation (Fig 5) in the Mediterranean. From these data, the effective population size was estimated at approximately 850 for the Mediterranean blue shark population (parametric 95% confidence interval: 450–1480).

### Genetic differentiation between Mediterranean and Atlantic blue shark

#### Haplotype distribution and population structure

**Fig 6:**
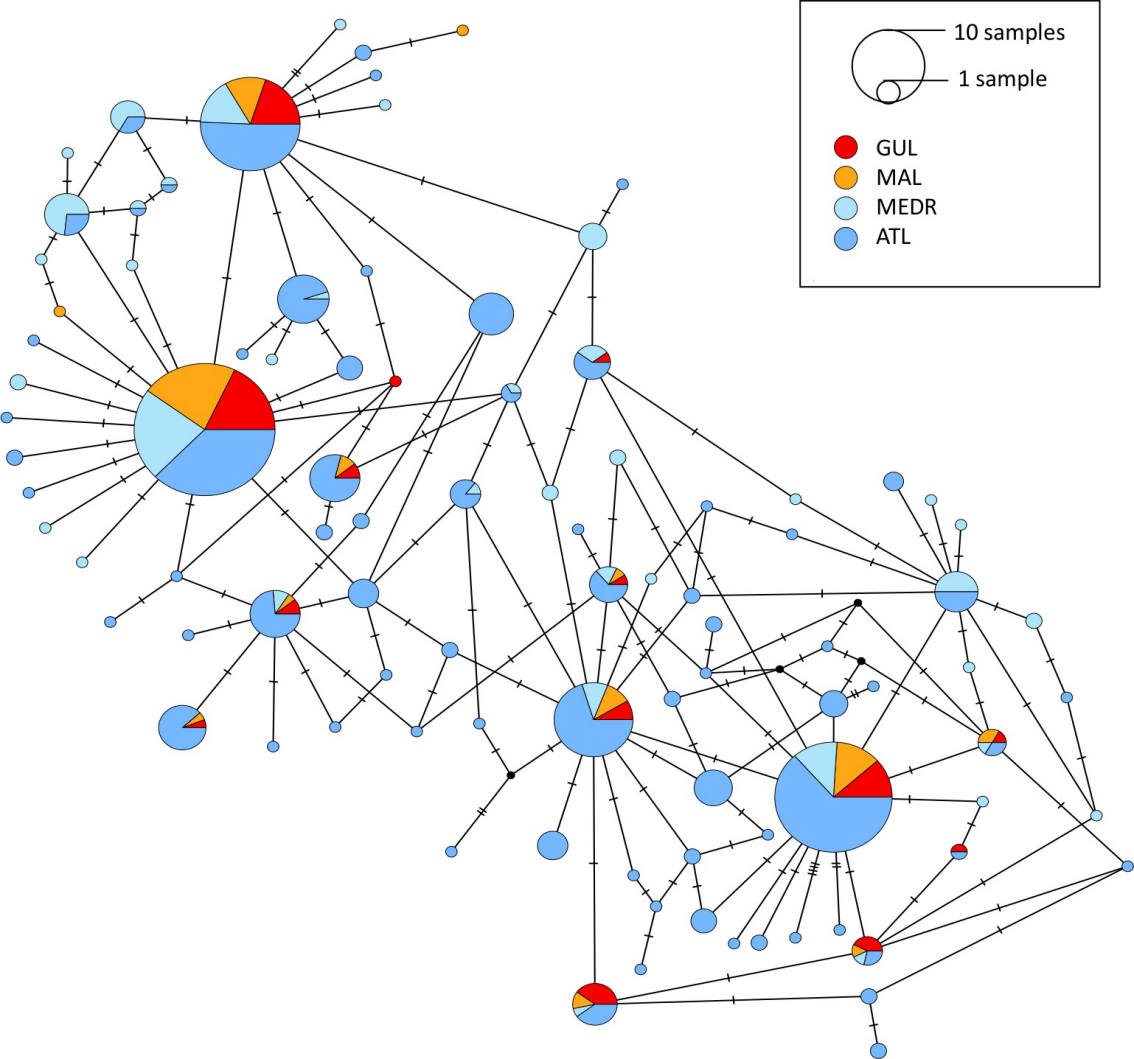
Mitochondrial control region haplotype network (Median Joining Network) based on Mediterranean and Atlantic blue shark sample collections. The network combines haplotypes from this study (MAL: Malta, orange; GUL: Gulf of Lion, red) and from GenBank (MEDR: remaining areas of the Mediterranean, light blue; ATL: Atlantic, blue). Black circles indicate inferred haplotypes (i.e. not observed).

Among the sixteen haplotypes recovered from MAL and GUL samples, 2 were recorded for the first time in the Mediterranean Sea (MED) and were never found in the Atlantic Ocean (ATL). When combining data from 4 studies, a total of 95 haplotypes were found in the Mediterranean Sea and the Atlantic Ocean out of 701 individuals. The majority of abundant haplotypes were shared within MED and ATL with similar frequencies and no evidence of differential spatial distribution (Fig 6). However, higher haplotype diversity was found in the Atlantic compared to the Mediterranean (‘MED’: this study plus published sequences), with 72% of Atlantic haplotypes being absent from the Mediterranean Sea (Nh = 73; N = 457 individuals). Nonetheless, 47% of Mediterranean haplotypes were also absent from the Atlantic (Nh = 44; N = 315 individuals), which indicates some degree of genetic isolation and limited gene flow between the two basins. Additionally, both the fixation index F_st_ and the pairwise distance φ_st_ differed significantly from zero (F_st_ = 0.0373; 95% confidence interval: 0.0149–0.0556 / φ_st_ = 0.0350; p_value < 0.001) indicating significant genetic differentiation between the Atlantic Ocean and the Mediterranean Sea.

#### Genetic diversity and demographic history

Genetic diversity calculated from mtDNA sequences in the MED sample (h = 0.887 ± 0.016, π = 0.00355 ± 0.00012) was significantly lower than in the ATL sample (h = 0.987 ± 0.003, π = 0.00532 ± 0.00018) regarding both haplotype (h) and nucleotide (π) diversities.

The coalescent analysis of the demographic history of blue sharks from the Mediterranean (MED) showed a population increase (Fig 4B) that started approximately 0.15Mya but was not significantly detected by Fu and Li’s F and D tests. In the Atlantic (ATL), a population increase was also observed and started earlier, approx. 0.4Mya (Fig 4C). This expansion was detected by Fu and Li’s F and D test with significantly negative values for the Atlantic basin (F = -2.60, D = -2.74, p-value <0.05).

## Discussion

### Genetic differentiation between Atlantic and Mediterranean blue sharks

#### Population structure

The Mediterranean blue shark population appeared as an isolated subgroup of the Atlantic population, with limited gene flow between the two areas. Significant F_st_ and φ_st_ indicate a genetic differentiation between the two populations. The haplotype network showed a greater diversity of haplotypes from the Atlantic, and a majority of Mediterranean haplotypes were also found in the Atlantic (53%) when the opposite is not true (28%). In addition, a significant part of the haplotypes was specific to the Mediterranean (47%), which shows a certain degree of recent differentiation between the two zones with limited gene flow from the Mediterranean to the Atlantic population. These elements tend to show that colonisation of the Mediterranean Sea by the Atlantic population has occurred, but that exchanges became limited between the two zones, which has led to the differentiation of haplotypes that are now found only in the Mediterranean. If there are exchanges, they are also mainly in the ‘Atlantic to Mediterranean’ direction as the number of haplotypes specific to the Mediterranean was important and was not found in the Atlantic. This is consistent with the tagging studies of blue sharks that, despite a low recapture rate, have shown no evidence of blue shark migration between the Atlantic and the Mediterranean [57–61]. Other pelagic or migratory fishes also exhibit genetic differentiation between Atlantic and Mediterranean populations at microsatellite and mtDNA loci, such as the meagre (*Argyrosomus regius*) [62] and the strait of Gibraltar serves as a barrier to gene flow for many species regardless of their spatial ecology [63].

#### Demographic history and effective population size

The coalescence analysis on the mtDNA gene showed a constant expansion of the populations of blue sharks both in the Atlantic and in the Mediterranean, the latter beginning more recently (0.15 Mya versus 0.4 Mya). These results are concordant with those of Leone et al. [24] for the Mediterranean Sea. The analyses of the demographic history of populations are mostly affected by the last transforming event that tends to mask any previous phylogenetic signals [64]. Thus, in the Mediterranean Sea, the signal of expansion around 0.15 Mya seems linked to the Riss-Wurm interglacial (previous to last glacial episode, 0.130–0.115 Mya) whereas the last glacial episode of the Holocene (0.115–0.011 Mya, LGP Last Glacial Period) does not seem to have affected the evolution of the genetic diversity of the blue shark in the Mediterranean. In the Atlantic, which represents a much larger body of water, the Mindel-Riss interglacial (0.42–0.3 Mya) seems to have mainly affected the diversity of the species, whereas the subsequent interglacial events had no significant effect. Contrasting population expansion timescales between the Atlantic and Mediterranean populations also occurred in the pelagic swordfish (*Xiphias gladius*) [65].

Similar to the demographic history, the contemporary effective population size (CNe) of the blue shark was also different between the two basins. Despite the same estimation method (linkage disequilibrium method), the effective size of the Mediterranean blue shark (CNe = 850), was fivefold lower than the one of the Atlantic population (CNe = 4500) [1] or the Pacific blue shark population (CNe = 5000) [20]. If genetic panmixia occurs, Ne is expected to be similar across the population range [1,66] therefore such a difference between Atlantic and Mediterranean blue shark effective population sizes also emphasizes the limited gene flow and the genetic differentiation between the two populations.

All these results argue for a genetic barrier between the Atlantic and Mediterranean populations, documented in many other species including pelagic migratory species including sharks, whales, dolphins, and swordfish [67,68]. However, they contrast with the results of Leone et al. [23,24] who argued for some degrees of connectivity between nurseries in the eastern Atlantic and the western Mediterranean, based on mitochondrial and SNP markers amplification on 207 individuals. Although a weak genetic structure was detected, they concluded that sufficient migration between the two spatially separated zones occurred, allowing the near-panmixia across the range. On the other hand, Bailleul *et al*. [19], using 200 samples and simulations, argued that the apparent lack of structure in the blue shark populations may be due to a lack of detection power of the fixation index F_st_ regarding recent population changes. Here, however, we detected a genetic differentiation between the Atlantic and Mediterranean blue shark populations using F_st_ and 701 mtDNA sequences, combining existing and new sequence datasets. This is in accordance with a recent genome-wide study using SNPs, which also found a significant genetic differentiation between the Northern Atlantic Ocean (n = 75) and the Mediterranean Sea (n = 54) [25]. Tagging data in both the Atlantic Ocean [57,58] and the Mediterranean Sea [59,60] confirm the absence of connectivity between the two basins so far, although additional tag deployment near the strait of Gibraltar would help determine any migration rate between the Mediterranean and adjacent Atlantic, which appears relatively complex with limited and probably unidirectional exchanges.

### Genetic characterisation of the blue shark within the Mediterranean Sea

#### Population structure within the Mediterranean Sea

No significant genetic differentiation between Malta and the Gulf of Lion was found with the Bayesian analysis of STRUCTURE. The levels of genetic diversity were also homogenous between the two locations, no distinct group emerged from the PCoA analysis, and the value of G_ST_ was not significantly different from zero. These results indicate that blue sharks in these locations form a single population. Leone et al. [24] showed a weak genetic differentiation between blue sharks in the Eastern basin and the Western basin within the Mediterranean, using SNPs and mitochondrial DNA markers. Such population structure pattern has been observed on other species such as the sea bass, *Dicentrarchus labrax*, [69] and the Atlantic bluefin tuna [70]. Considering this result, our study shows that blue sharks occurring off Malta are related to the population in the Western basin. However, with satellite tracking data on 39 blue sharks from the Western Mediterranean basin, Poisson et al. [60] did not observe any migration further than the Strait of Sicily. They proposed a migration pattern where some large juvenile females migrate north-eastward from the Alboran Sea to the Balearic Sea and the Gulf of Lion, while others follow the Algerian current towards Tunisia and then move northward in the Tyrrhenian Sea between Sicily, Sardinia, and Italy. This putative migration pattern excludes Malta, thus our study does not fully support it. Some sharks may enter the Sea of Sicily with the Algerian current flowing southward along the coast of Tunisia. Whether they continue their migration further East is currently unknown, and tag deployment in this area as well as additional genetic studies between the Western and Eastern basins remain necessary to unravel the blue shark population structure within the Mediterranean fully. Notably, migration patterns of adults and males still lack supportive data and require specific attention [60].

#### Low contemporary effective size in the Western Mediterranean population

The contemporary effective size was equal to approximately 850 (95% confidence interval: 450–1840) in the Mediterranean, which is fivefold lower than the adjacent Atlantic and the Pacific [1,20]. This result is surprising regarding the remarkably high fecundity of the blue shark (30 pups on average [13]). In comparison, the effective population size of the sandbar shark, *Carcharhinus plumbeus*, in the North-West Atlantic is about 1500 [71], when this shark is known to have lower fecundity (8 pups on average) than the blue shark [72]. That of the smalltooth sawfish, *Pristis pectinata*, one of the most endangered sharks in the world whose abundance has declined by 95% during the past 50 years [73], lies between 250 and 500 in the same region [74]. The low effective size of the Western Mediterranean population highlights and confirms the vulnerability of the blue shark in this area. Despite a high reproductive capacity, few individuals may survive and participate in the transmission of genetic heritage, and the sustainability of the population may be more threatened than its fecundity alone suggests. In addition, Pinsky *et al*. [75] suggest that an effective size greater than 3000 is needed to limit the risk of loss of genetic diversity under overfishing pressure. In the Mediterranean, where a population decline of 90% is estimated based on catch data [18], the effective size is far below. This is not sufficient at this stage to maintain a stable level of genetic diversity under the current fishing pressure [76] and thus this threatens the long-term resilience of the population in the Mediterranean Sea [77].

#### The Gulf of Lion constitutes a nursery for the species

While no genetic differentiation was found between blue sharks from the Gulf of Lion and Malta, a major difference in size distribution was observed. All blue sharks caught in the Gulf of Lion were juveniles, 14% of which were less than one year old. On the contrary, in Malta, 76% were adults. This finding on the relative abundance of young-of-the-year sharks (YOY) supports the recent conclusions of Poisson et al. [60] drawn with satellite tracking of large juvenile females and confirms that the Gulf of Lion constitutes a nursery ground for the species in the Western Mediterranean basin. Heupel *et al*. [78] developed a systematic approach to identify nurseries based on three criteria:

- YOY are more abundant than in other areas.
  The proportion of YOY in the Gulf of Lion is higher than in Malta. Although the type of fishing has been suggested to explain the size difference in catches (recreational vs commercial fishing [24]), Megalofonou et al. [16], with a sampling method comparable to the one used in Malta (records on board longline vessels and at the main fishing ports), found 11% (N = 870) of YOY sharks in the Adriatic, Ionian, and Aegean Seas and the Levantine basin. This percentage is comparable to the 14% found in the Gulf of Lion. The abundance of YOY sharks in the Gulf of Lion is therefore similar to those in areas where nurseries were previously defined for the Mediterranean Sea.
- juvenile sharks tend to remain or return to the area for extended periods.
  While 14% of individuals were YOY, our study could not determine whether the blue sharks remain or return to the Gulf of Lion, due to a low sampling size, which decreases the probability of recapture. Tracking data of blue sharks in the Mediterranean currently excludes young-of-the-year and small juveniles due to the technical challenge of tagging the smallest individuals [60]. This technical issue should be addressed in the future, as tracking data of young-of-the-year would better address this criterion. Tracking of juvenile thresher sharks *Alopias vulpinus*, another highly mobile and pelagic species, demonstrated their use of open coastal habitat over the continental shelf as a nursery in California [79]. Nonetheless, juvenile blue sharks are thought to remain in coastal waters and not take part in extensive migrations before reaching a size of 130cm (approx. 2–3 years old) [13,80]. Additionally, a global meta-analysis of foraging habitat suitability for different size classes of blue sharks showed that the Western Mediterranean including the Gulf of Lion is a suitable foraging habitat for small juvenile blue sharks throughout the year [81]. These sharks are thus likely to remain in the Gulf of Lion for the first years of their life.
- the area has been repeatedly used over the years.
  YOY and juvenile sharks were sampled repeatedly in the Gulf of Lion over 6 years (2012–2018) and are still observed and sampled to date.

Blue shark nurseries were earlier identified in the Adriatic Sea for the Mediterranean Sea [16], and off the Azores, the Iberian Peninsula [82], South-West South-Africa, South-East Brazil [80], and in the Central North Atlantic [17] for the Atlantic Ocean. Other pelagic sharks are also known to use open areas as nurseries, such as the thresher shark [79], and the great white shark [83]. The difference in size distribution consistent over the years along with recent modelling and satellite tracking data confirms that the Gulf of Lion constitutes a nursery for the blue shark, which highlights its important ecological role for the species.

#### Evidence of sibling aggregation

Interestingly, the Bayesian clustering in STRUCTURE and the parentage analysis in Colony revealed 4 clusters of 10 full siblings occurring in the Gulf of Lion and Malta (Table 2). Given our low sampling size, such a high number of pairs is surprising and might be evidence of sibling aggregation. Aggregation and schooling are common shark behaviours and may be driven by food abundance or confer protection from predators [78,84]. Most often, sharks aggregate by species, size, or sex [85]. Particularly, adult and sub-adult blue sharks are known to segregate by sex and size [13]. In nurseries, lemon sharks, *Negaprion brevirostris*, form size-driven aggregations that confer anti-predatory and foraging advantages [86]. While it is known that teleost fishes and marine mammals are capable of social recognition [87–89], kinship-driven aggregations in sharks have been widely overlooked to date. Kinship may be playing a role in the aggregation of juvenile lemon sharks [86]; and small spotted catsharks, *Scyliorhinus canicula*, have social preferences for familiar sharks but not necessarily for relatives [90]. Our finding highlights the need for further investigation into potential kinship-driven social aggregations of blue sharks.

### Use of genetic data for management and implications for the blue shark in the Mediterranean

#### Genetic parameters inform on species’ long-term vulnerability

Genetic diversity is considered an important parameter to inform the conservation status of a species because its long-term survival depends on it [6,77]. As the blue shark is listed as Critically Endangered on the IUCN Red List in the Mediterranean [18], we would thus expect to observe a lower genetic diversity in this basin. However, this is not the case, and no clear pattern appears from the comparison with other basins. Similarly, the smalltooth sawfish (*P*. *pristis*) population of the North-West Atlantic also shows a high genetic diversity (Ho = 0.43–0.98) despite its sharp population decline and critically endangered status [74]. Pinsky *et al*. [75] suggested that the loss of diversity due to a steep decrease in population size could take as many as seven generations to be detected. The abundance of blue sharks in the Mediterranean has decreased by 78% to 90% in three generations [18]; the loss of genetic diversity may thus be only visible in four more generations, i.e. approximately 30 more years. This highlights that genetic diversity indices should be used with caution when applied as a proxy for conservation status, especially for species that are undergoing a high rate of population decrease. On the contrary, the contemporary effective population size indicated a clear pattern of genetic vulnerability in the Mediterranean compared to other basins, which is following IUCN status. This parameter may thus be more informative than the genetic diversity to reflect recent population changes.

#### Implication for blue shark management in the Mediterranean

Our study supports the distinction of two genetic stocks for the blue shark in the Mediterranean Sea and Atlantic Ocean. Recent important tagging and tracking efforts both in the Atlantic [61] and the Mediterranean Sea [60] also support a lack of connection between the two basins. Thus, the precautionary principle applied by the ICCAT and consisting of separating these basins for management purposes is appropriate [22]. In the Mediterranean, the Western and Central basins may form a unique stock and faces a high risk of extinction not only on a short but also on a long timescale. Management and conservation measures should consider this new genetic insight that strongly comforts the IUCN status of this regional population as Critically Endangered. As of now, no fishing regulation is enforced in the Mediterranean, and the fishing pressure is overall particularly high in the Gulf of Lion [91]. The report of the ICCAT 2023 regarding blue shark fishing pinpointed a lack of reported catch data (landing and dead discards) for this species in the Mediterranean and a lack of improvement in the recovery of these data over recent years [22,92]. The ICCAT strongly encourages reporting catch data in order to provide an up-to-date stock assessment, and we recommend taking additional efforts in management and regulation.

The Eastern Gulf of Lion and the canyons of Costa Brava have just been recognised as an area of importance for the blue shark (Important Shark and Ray Areas) due to the presence and movement of the species. We suggest extending this recognition to the Western Gulf of Lion both because of its nursery role and because of the possibility of surveying the Western Mediterranean population while sampling juveniles from this area. Additionally, the Gulf of Lion is the coldest region in the Western Mediterranean and could become increasingly important for the species in the context of global warming. Increasing temperatures may trigger a distribution shift towards cooler waters [93] and impact juveniles’ survival on the nursery ground [60]. Particular management efforts and monitoring of environmental conditions should be deployed in the Gulf of Lion to ensure the survival of juvenile blue sharks as their survival rate is crucial for the growth rate of the population [60,94]. Additionally, the deployment of tagging and capture-release-recapture programs is still needed to study the residency rate of Young Of the Year individuals and confirm the second criterion proposed by Heupel *et al*. [78]. Finally, this study proves the ability of Citizen Science, as well as catch-and-release fishing, to provide valuable data for conservation research and management.

## Supporting information

S1 Fig Sampling locations of blue sharks in the Atlantic Ocean and Mediterranean Sea from all the studies used for comparison in this research. Ferrette et al., unpublished (n = 108), Leone et al., 2017 (n = 170), Veríssimo et al., 2017 (n = 273), this study (n = 150). The map was created using the R software and the publicly available map dataset *Natural Earth*.
(TIF)

S2 Fig Bayesian clustering of blue shark individuals from STRUCTURE analysis before removing the full siblings from the analysis and showing the different full-sib clusters. a) Bar plots for K from 2 to 4. Each individual is represented by a vertical bar partitioned into coloured sub-bars whose lengths are proportional to its estimated probability of membership for the K clusters. 1: individuals from Malta, 2: individuals from the Gulf of Lion. Individuals belonging to the full-sib clusters identified with Colony (Table 2) are indicated. In each K, runs may inconsistently highlight one cluster or another, therefore the number of runs in each K for which the bar plot configuration appears is indicated in brackets. b) Plot of the mean of estimated “log probability of data” for each value of K. c): DeltaK of Evanno’s method based on the rate of change in the log probability of data C).
(TIF)

S3 Fig Principal Coordinates Analysis (PCoA) showing the genetic distance of all blue shark samples from the Gulf of Lion and Malta. Red dots represent samples from the Gulf of Lion, orange diamonds represent samples from Malta. a) PCoA for Axis 1 vs Axis 2. b) PCoA Axis 2 vs Axis 3. The percentage of variance for each axis is indicated in brackets.
(TIF)

S1 Table List of sampled individuals of blue shark Prionace glauca included in this study, with associated total length (cm), sex, maturity, sampling region, GPS coordinates and sampling year. (XLSX)

S2 Table Composition of primer mix in each multiplex created for microsatellite amplification. The temperature corresponds to the annealing temperature, and the volume (given in μL) corresponds to the volume used for each forward primer at an initial concentration of 100μM. The same volume was used for the reverse primers.
(PDF)

S3 Table List of blue shark’s Dloop mitochondrial genes available on GenBank and used in this study. (PDF)

S4 Table Complete genotype matrix of all individuals genotyped for this study. (XLSX)
