# Supplementary figures and images for: New insights into population structure, demographic history, and effective population size of the critically endangered blue shark Prionace glauca in the Mediterranean Sea
Source: PLoS One. 2024 Jun 17;19(6):e0305608. doi: 10.1371/journal.pone.0305608 (PMC11182550; doi:10.1371/journal.pone.0305608)

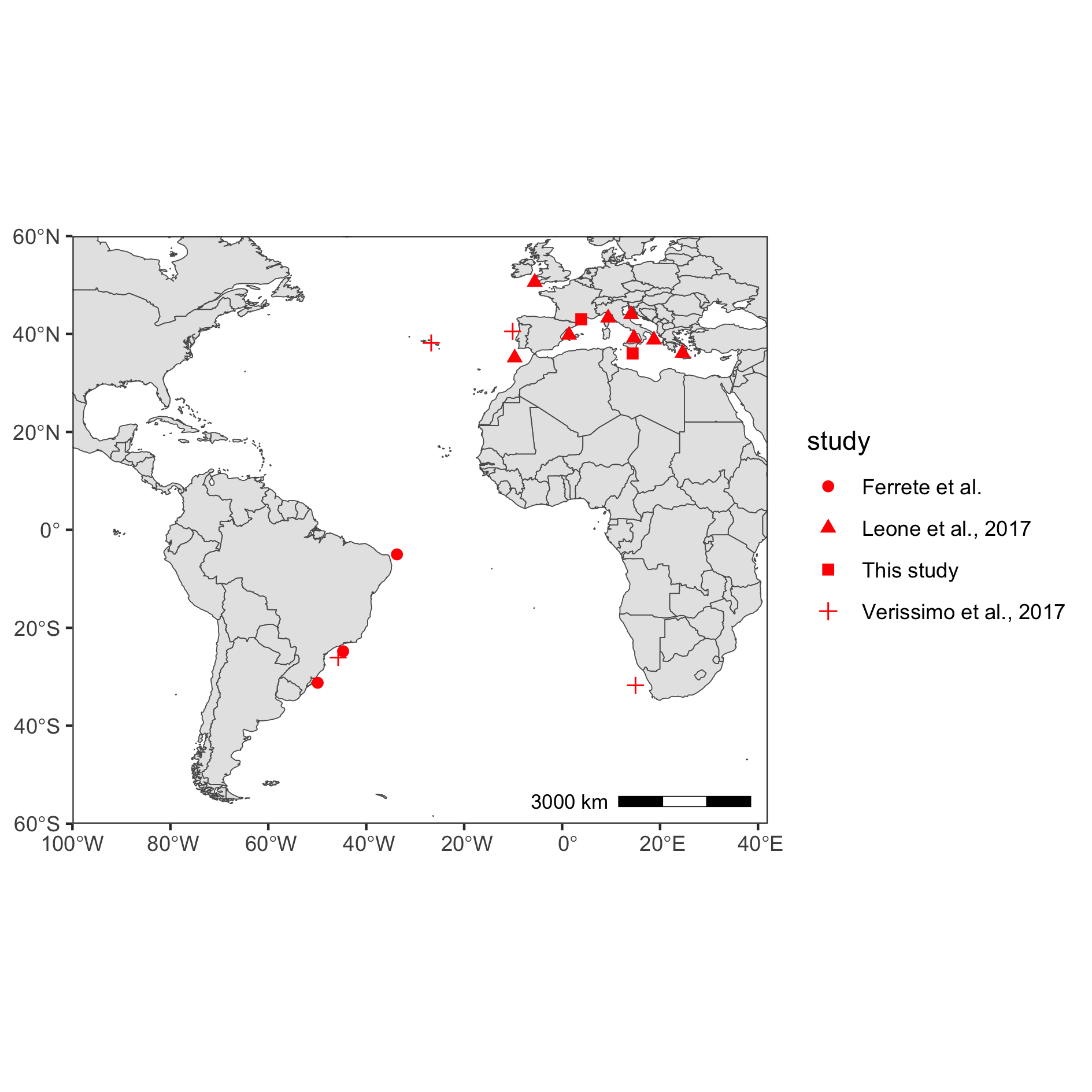

Supplement: S1 Fig — Ferrette et al., unpublished (n = 108), Leone et al., 2017 (n = 170), Veríssimo et al., 2017 (n = 273), this study (n = 150). The map was created using the R software and the publicly available map dataset Natural Earth. (TIF) [file pone.0305608.s001.tif]

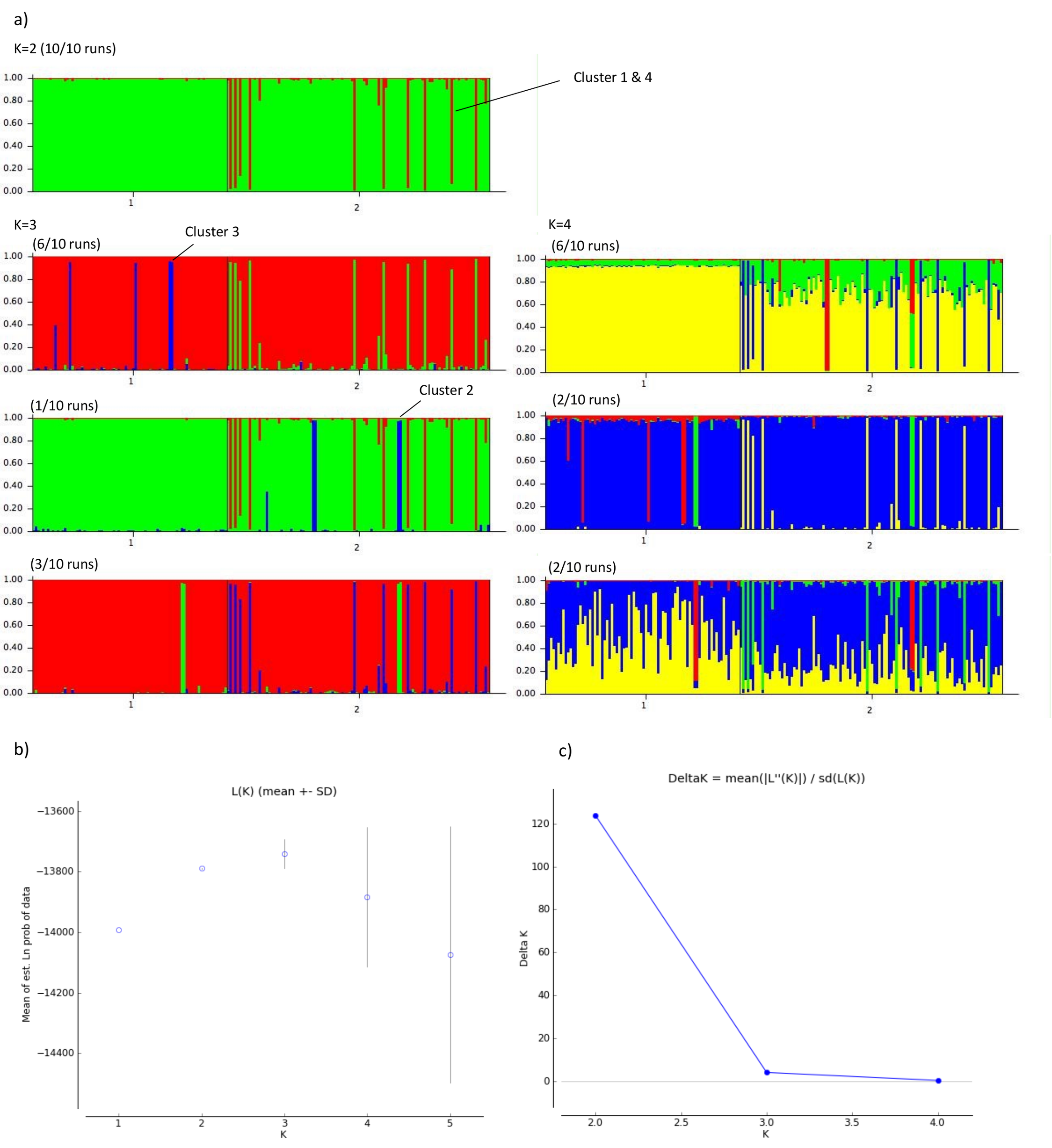

Supplement: S2 Fig — a) Bar plots for K from 2 to 4. Each individual is represented by a vertical bar partitioned into coloured sub-bars whose lengths are proportional to its estimated probability of membership for the K clusters. 1: individuals from Malta, 2: individuals from the Gulf of Lion. Individuals belonging to the full-sib clusters identified with Colony (Table 2) are indicated. In each K, runs may inconsistently highlight one cluster or another, therefore the number of runs in each K for which the bar plot configuration appears is indicated in brackets. b) Plot of the mean of estimated “log probability of data” for each value of K. c): DeltaK of Evanno’s method based on the rate of change in the log probability of data C). (TIF) [file pone.0305608.s002.tif]

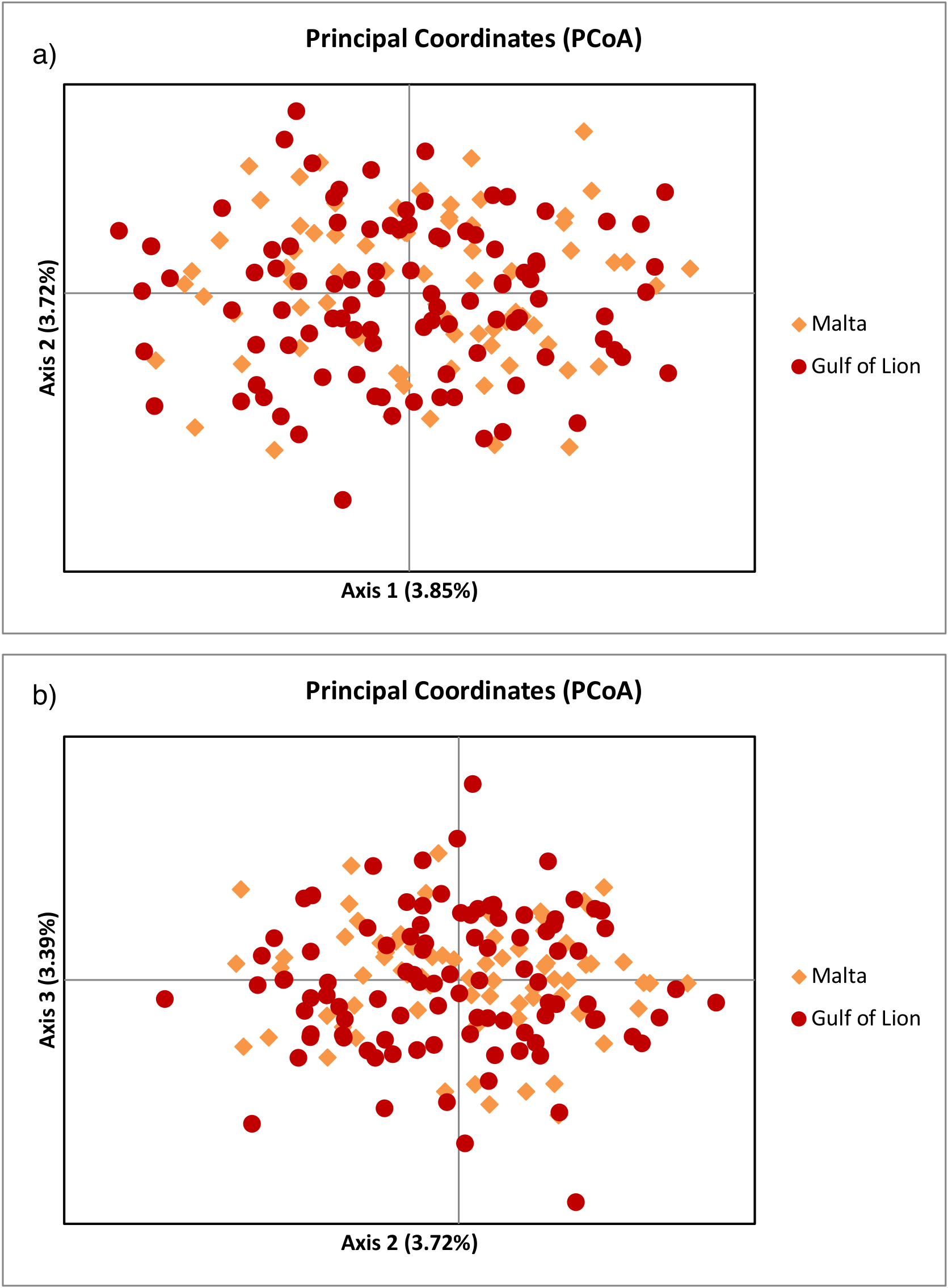

Supplement: S3 Fig — Red dots represent samples from the Gulf of Lion, orange diamonds represent samples from Malta. a) PCoA for Axis 1 vs Axis 2. b) PCoA Axis 2 vs Axis 3. The percentage of variance for each axis is indicated in brackets. (TIF) [file pone.0305608.s003.tif]
