# Supplementary material for: New insights into population structure, demographic history, and effective population size of the critically endangered blue shark Prionace glauca in the Mediterranean Sea
Source: PLoS One. 2024 Jun 17;19(6):e0305608. doi: 10.1371/journal.pone.0305608 (PMC11182550; doi:10.1371/journal.pone.0305608)
Supplement: S2 Table — The temperature corresponds to the annealing temperature, and the volume (given in μL) corresponds to the volume used for each forward primer at an initial concentration of 100μM. The same volume was used for the reverse primers. (PDF) [file pone.0305608.s005.pdf]

S2 Table

**Multiplex 1: 63°C**	**Multiplex 2: 60°C**	**Multiplex 3: 57°C**	**Multiplex 4: 57°C**	**Multiplex 5: 53°C**
Pgla02 : 0.010	Pgla03 : 0.005	Pgla06 : 0.008	Pgla07 : 0.008	Pgla04 : 0.010
Pgla05 : 0.010	Pgla09 : 0.010	EFQGD : 0.010	BEF94 : 0.010	EHDO8 : 0.012
Pgla08 : 0.010	D0MST : 0.008	CY92Z : 0.008	D1Q4A : 0.012	EJABW : 0.005
A2ASY : 0.012	TB02 : 0.010	FV6T5 : 0.008	EWU1E : 0.012	TB01 : 0.010
DAE7L : 0.008	TB15 : 0.015	DZONX : 0.008	TB04 : 0.010	TB13 : 0.010
