# Supplementary material for: New insights into population structure, demographic history, and effective population size of the critically endangered blue shark Prionace glauca in the Mediterranean Sea
Source: PLoS One. 2024 Jun 17;19(6):e0305608. doi: 10.1371/journal.pone.0305608 (PMC11182550; doi:10.1371/journal.pone.0305608)
Supplement: S3 Table — (PDF) [file pone.0305608.s006.pdf]

S3 Table

GenBank Accession #	Reference	Origin
KY923141 to KY923192	Veríssimo *et al.* (2017)	Atlantic Ocean
KY994016 to KY994042	Ferrette *et al.* (not published)	Atlantic Ocean
MH085076 to MH085080		Atlantic Ocean
MH806840 to MH806841		Atlantic Ocean
MG545809 to MG545841	Leone *et al.* (2017)	Atlantic Ocean
MG545888 to MG545893		Atlantic Ocean
MG545732 to MG545808		Mediterranean Sea
MG545842 to MG545887		Mediterranean Sea
MG545894 to MG545901		Mediterranean Sea
